# The Study of the Materia Medica

**Published:** 1881-03

**Authors:** 


### THE STUDY OF THE MATERIA MEDICA.

UNDOUBTEDLY the most perplexing problem presented to the student of medicine is the Materia Medica. His first idea on opening one of the manuals is that each drug has produced all the aches and pains which human flesh can ever feel. To him it seems useless to study more than one such comprehensive remedy, and perforce, one small bottle will contain all the armentarium he will need to conquer any and every disease.

At first glance the student is appalled at the multiplicity of symptoms each drug has produced; to him, one drug seems to require a life's study to clear up its therapeutic chaos. When, therefore, he is required to memorize hundreds of such drugs and to diagnose each from the rest, he deems the task beyond human accomplishment. Many practitioners lazily prefer routine prescribing, or worse allopathic dosage, to attempting this task. Its alleged impracticability being used as a cloak for this laziness.

To practice homœopathy one must find the simillimum remedy for each and every case; to do this he must individualize his drugs. That is, he must not only know the characteristic symptoms each drug has produced, but he must also know how these differ from similar symptoms of other drugs. Those who are too lazy to do this, resort to alternation of drugs or to palliatives, quinia, etc.; thus violate a tacit pledge and endanger life.

The Materia Medica is certainly a difficult study; it is a life study, but it can *be mastered* as far as is practically necessary. This has been done, and any man of average ability should be ashamed to acknowledge he can not do what others have done. In this work repertories are a great, and should be an ever-present, assistance. Yet they do not lessen the necessity for continual critical study of the Materia Medica.

The physician who has not studied and compared his remedies in the quiet of the study will scarcely be able to do so satisfactorily in the hurry and excitement of the sick-room. It is then indispensably necessary for the physician who wonld select his remedies with certainty, that he carefully study them *beforehand* and fix in his mind the varied symptoms which each produces and the differences between similar symptoms of different drugs. It is this comparison and individualization of drugs which simplifies and lightens our therapeutic task and clears up many a seeming contradiction. It is also by comparison that the true characteristic symptoms of each drug becomes apparent. If those who so loudly complain of the worthlessness of our Materia Medica, would study it more, perhaps they would find it more useful. But *instead* they close their books and resort to palliation and such-like, while waiting for *others* to perfect the Materia Medica. There are three points in which the student is apt to be puzzled in his study of the Materia Medica. These will now be considered.

1. Where a drug has two apparently contradictory symptoms, *one* of which is characteristic. Such as aggravation and amelioration from same cause; or both constipation and diarrhœa, and so on. Thus *Arsenic* has aggravation and amelioration from lying in bed; this appears contradictory until it is remembered that *Arsenic* is aggravated by *rest* in bed, and ameliorated by the warmth of bed. *Mercurius* presents the same apparent anomaly, under opposite conditions, being also agg. and amel. by lying in bed, but here the agg. is from the heat and the amel. from the rest.

Again under *Sepia* we read "aggravation (of headache) from least motion;" in another place we find "continued hard motion relieves headache." This we know is a peculiarity of *Sepia*, that a slight, easy motion aggravates, while violent motion, such as skating or dancing, alleviates. A somewhat similar peculiarity is that of amelioration from hard pressure, while a mere touch or slight pressure aggravates pain. This symptom occurs most prominently under *bell.*, *china*, and *nux vom.*

Many drugs have opposite conditions, such as constipation and diarrhœa, great thirst or thirstlessness, etc.; these are alternatives, one being the primary, the other the secondary effect. One is generally characteristic of the drug; the other less important. Thus, *arsenic* has prominently great thirst, wants to drink little and often, yet it also has thirstlessness; *pulsatilla* has generally thirstlessness, but it has also great thirst. Now great thirst is characteristic of *ars.*, while thirstlessness is just as characteristic of *puls;* the opposite conditions—lack of thirst for *ars.* and great thirst for *puls.*—being the unimportant, or less characteristic, symptoms.

Again, *opium*, the time honored remedy of allopathy for diarrhœa, causes constipation when used in massive doses, as its primary effect; while later action produces diarrhœa. Thus, while allopathic empiricism found *opium* useful in bowel complaints, only as a check for diarrhœa, homœopathic law shows it to be useful for both diarrhœa and constipation, An example, furnishing a striking rebuke to those who claim that our law *restricts.*

2. Another difficulty for the student is where two or more remedies have the same symptom, and equally characteristic, differing only in character or in concomitants. How to diagnose, how to tell when a symptom, shared by many drugs, is indicative of one, is *the* question. This difficulty can always be met in practice, by comparing the characteristic symptoms of the case to be cured, with those characteristic of the drug. Not one symptom, nor yet two, but the totality—the totality of the characteristic and peculiar symptoms.

In studying the Materia Medica we may learn to diagnose similar symptoms of different remedies by studying their concomitants or by the conditions under which they appear or are influenced. Thus, to illustrate, both *rhus* and *pulsatilla* have restlessness and under both it is very characteristic. In both the patient *must* move though it pains him to do so. Thus far they are alike; but the *rhus* patient finds *momentary* relief from this change, the *puls.* patient finds *none.* A *rhus* patient is improved by slow continued motion (though worse on commencing to move), out doors if not *cold;* the *puls.* patient is improved by slow motion out doors if it does not *heat.*

Another illustration is found in the *puls.* and *sulph.* desire for air, or dislike for a too close room; both want door or window opened. Under *puls.* this occurs often in labor or other trouble foreign to chest; under *sulph.* it is from a respiratory trouble. *Arsenic* and *puls.* have a similar thirst. The *arsenic* thirst is, for small and frequent drinks, caused by a great burning, while *puls.* patient desires to moisten, or cleanse, the dry, clammy mouth. *Arsenic* patient takes large amounts of water which he vomits *immediately; puls.* has at times great thirst for large quantities which are not thrown up. *Phosphorus* also has this great thirst, but here the water is not vomited until it becomes *warm* in the stomach. *Silicea* and *magn. mur.* have a headache better from wrapping head up warmly. The *silicea* pain is better in doors and from rest, while the *magn. m.* pain is better from exercise in open air; in fact, compels one to walk about, and is worse when lying.

*Ant. cru., ant. tart.*, and *cina*, patients don't want to be touched; they are not sore, nor does touching hurt them; it is peevishness. While *bell., arn.*, and *china*, patients are so sensitive, from pain or soreness, that they don't want to be touched or even for one to come *near* them, for fear of rough handling.

3. A third difficulty is where a symptom occurs under two drugs (or more), but is characteristic of *one* only. In the case just illustrated the symptom was found under two or more drugs, but there it was characteristic of *both*, as (the instance there given) the restlessness of *rhus* and *puls.*

In illustration of this point we may mention the weak, empty feeling in abdomen found under *phosphorus;* other drugs (*merc., sanguin*, etc.) have this symptom, but it is most prominent under *phosphorus.*

Most of those prominently characteristic symptoms, commonly known as "key-notes," come under this head. Nearly all of these "key-notes" are found under more than one drug but they are generally characteristic of one only. Take, for instance, the great desire of children to be carried on the arm, which is so characteristic of *chamomilla*. This is found under *ant. tart.*, *ars.*, *cina*, *ignat.*, *kali c.*, and *puls.* *Aconite* has amelioration from being rocked or carried fast; *cina*, also, has amelioration from being rocked; *ars.* wants to be carried fast, *puls.*, slow. *Bromium* wants to be carried fast on account of dyspnœa, often indicated in croup.* These prominent symptoms are useful as guide-posts pointing out the right path; which path, if followed, may lead to the right remedy.

But one must be cautious not to prescribe from one single indication; still less should the careful physician prescribe one invariable remedy every time a key-note crops out. *There is no one symptom which invariably indicates the one remedy.* Each case of sickness has its peculiar symptoms; each drug has its peculiar symptoms; these should be compared, and when a drug is found whose characteristic symptoms are similar to those of the disease, then, that drug is the simillimum. E. J. L.

---

### A GEM.

The following, for pure knavery, is a gem.

"*Theoretically* it may do to assert that homœopathic physicians must never give but a single remedy in a case of disease, but *practically* it is quite a different matter"!

Evidently Dr. H. C. Wood had this gem before him when he wrote, "For a man to practice homœopathy at present necessitates that he be either ignorant, foolish or knavish—that is if it be knavish to live a lie."

* For this, and many other indications, the writer has to thank Dr. Ad. Lippe.

